# SCIP/SIEA and PAP: The New Workhorse Flaps in Soft Tissue Reconstruction for All Body Regions

**DOI:** 10.3390/jcm14030921

**Published:** 2025-01-30

**Authors:** Alberto Franchi, Filippo Andrea Giovanni Perozzo, Cesare Tiengo, Jonas Walber, Alice Parisato, Abdul Rahman Jandali, Florian Jung

**Affiliations:** 1 Department of Hand and Plastic Surgery, Cantonal Hospital of Winterthur, Brauerstrasse 15, 8400 Winterthur, Switzerland; jonas.walber@ksw.ch (J.W.); alice.parisato5@gmail.com (A.P.); abdulrahman.jandali@ksw.ch (A.R.J.); florian.jung@ksw.ch (F.J.); 2 Department of Plastic, Reconstructive and Aesthetic Surgery, University Hospital of Padua, 35128 Padova, Italy; filippo.perozzo@gmail.com (F.A.G.P.); cesare.tiengo@unipd.it (C.T.)

**Keywords:** reconstructive microsurgery, SCIP flap, SIEA flap, PAP flap, free flap reconstruction, soft tissue defects, donor site concealment, perforator flaps, functional and aesthetic results, flap complication rates, surgical outcomes

## Abstract

**Background:** In reconstructive microsurgery, the aesthetic outcome has gained increasing importance, and new applications of flaps have been explored, focusing on improved donor site concealment. This paper presents our experience with flaps gaining popularity in reconstructive microsurgery, specifically the SCIP/SIEA and PAP flaps. **Methods**: Since August 2022, SCIP/SIEA and PAP flaps have been offered for soft tissue reconstruction across all body regions. These flaps were added to the other traditionally offered free flaps, such as RFF, mSAP, ALT, DIEP, and LD. Where the defect could be equally reconstructed using flaps from various donor sites, the choice of donor site was left to the patient. In all other cases, the donor site was selected by the surgeon according to clinical needs. This retrospective study analyzes the first author’s experience with the SCIP/SIEA and PAP flaps, providing an overview of their applications, outcomes, advantages, and disadvantages. **Results**: A total of 79 patients were reconstructed with 86 free flaps during the study period. The SCIP/SIEA flap was used in 54 patients, and the PAP flap in 18 patients. Flaps other than SCIP/SIEA were used in the remaining seven. Among the 27 patients who were given the option to choose their donor site, 8 selected either the abdomen or inner thigh (5 and 3 cases, respectively). The remaining 19 patients expressed no preference and left the choice to the surgeon. Defects involved the head and neck in 30 patients (38.0%), extremities in 25 (31.7%), the breast in 23 (29.1%), and the trunk in 1 patient (1.3%). Major complications occurred in 12 patients (15.2%) while minor complications managed conservatively occurred in 18 patients (22.8%). Four flaps (4.7% of all flaps) were lost. **Conclusions**: In our clinical practice, the SCIP/SIEA and PAP flaps have proven reliable as workhorse flaps for small to large soft tissue defects. For very large defects, the latissimus dorsi flap remains the most reliable solution.

## 1. Introduction

In recent years, reconstructive microsurgery has increasingly focused on achieving both functional and aesthetic outcomes at donor and recipient sites [1,2,3,4,5,6]. This shift reflects improvements in microsurgical techniques, which have led to more consistent flap survival and reliable functional results [7,8]. As these procedures evolve, patient expectations for aesthetic refinement have consequently increased, expecting their reconstructions to not only restore function but also provide an aesthetically pleasing result that supports social reintegration and enhances quality of life [9,10].

The radial forearm flap (RFF), anterolateral thigh flap (ALT), latissimus dorsi flap (LD), deep inferior epigastric artery perforator flap (DIEP), and medial sural artery perforator flap (mSAP) remain among the primary choices worldwide for the microsurgical reconstruction of soft tissue defects, depending on factors such as the location and size of the defect, the need for specialized tissue components, and the surgeon’s experience [11,12,13,14,15]. More recently, however, there has been a rise in the popularity of other perforator flaps, particularly the superficial circumflex iliac perforator (SCIP) flap and the profunda artery perforator (PAP) flap [16,17,18,19,20].

The superficial circumflex iliac artery perforator (SCIP) flap has emerged in recent years as a versatile option in reconstructive surgery that utilizes perforators from the superficial circumflex iliac artery, involving the harvest of thin, pliable skin from the groin area [21]. Its use has grown steadily [16], attributed to its straightforward dissection, low donor-site morbidity, and cosmetic benefits—especially since the donor site is well-concealed. The SCIP flap is particularly suited for resurfacing small to moderate-sized defects on areas such as the face, hands, or feet, where minimal bulk is desirable. 

**Figure 1:**
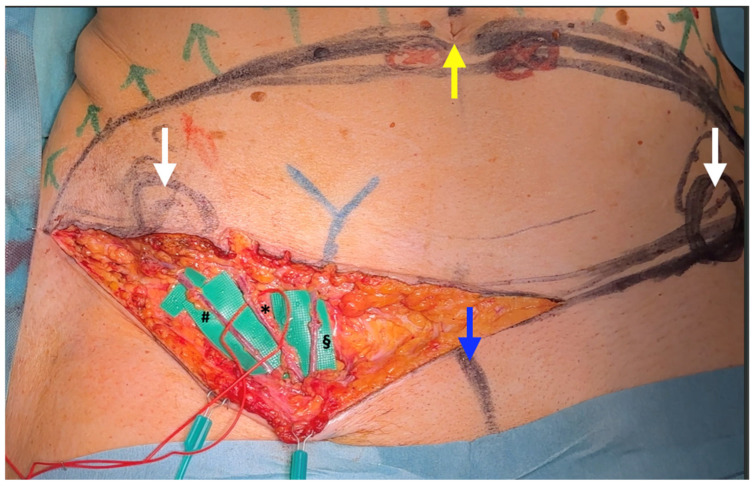
Vascular anatomy of the SCIP/SIEA flap. The artery and accompanying veins (*), as well as the SCIV (#) and SIEV (§), are shown emerging from the femoral triangle and proceeding cranially, superficial to the inguinal ligament. White arrows indicate the ASIS, the yellow arrow points to the umbilicus, and the blue arrow marks the midline just cranial to the vulvar cleft. The identified artery (*), according to the literature, may be referred to as either the SIEA or the “superficial branch” (or “medial perforator”) of the SCIA. Some authors classify this artery as a perforator, specifically a “direct” or “septo-cutaneous” perforator.

A key clarification on flap nomenclature is essential, however, to further elucidate this subject. In the available literature on the SCIP and SIEA flap, which we have meticulously reviewed, there is no consistent distinction between the artery referred to as the “superficial branch”, also known as the “medial superficial perforator” [22], and the artery commonly known as the superficial inferior epigastric artery (SIEA) (Figure 1) [23]. Furthermore, the definition of when a blood vessel should be classified as a “perforator” is widely debated in the literature, with some authors suggesting that the SIEA should be referred to as the SIEAP [24]. To simplify this complex issue, in this article we refer to the “SCIP/SIEA flap” as a flap based on an artery originating from the femoral artery, extending cranio-laterally and superficially over the inguinal ligament, toward the anterior superior iliac spine (ASIS), typically running slightly medial to it. In this study, SCIP flaps based on the so-called “deep branch” (or “lateral perforator”), which runs deeper and in contact with the sartorius muscle beneath the fascia lata [22,25,26,27] were not utilized.

**Figure 2:**
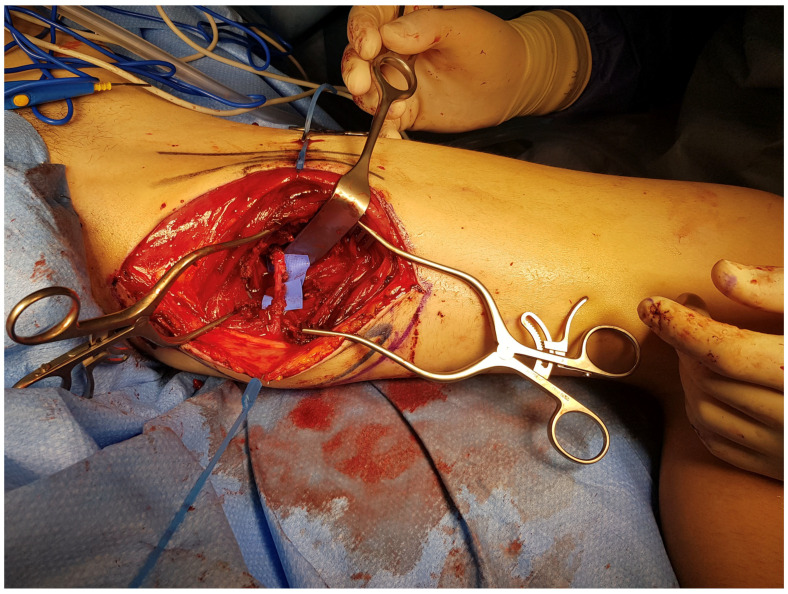
PAP flap harvesting. The postero-medial aspect of the left thigh is shown (with the knee on the right side and the groin crease on the left). The musculo-cutaneous perforator is dissected from the abductor magnus muscle and highlighted with a blue background.

Another emerging option in reconstructive surgery is represented by the profunda artery perforator (PAP) flap (Figure 2) [28]. Primarily used in breast reconstruction for patients who lack sufficient abdominal tissue, the PAP flap also serves as an excellent choice for those looking to avoid abdominal scarring [29]. Compared to the transverse upper gracilis (TUG) flap, its major advantage lies in muscle preservation, which helps reduce functional impairment at the donor site [30]. Furthermore, while the technique is more technically challenging, the PAP flap reliably achieves excellent aesthetic outcomes with discreetly concealed scars and low complication rates, such as fat necrosis and seroma [31]. High patient satisfaction, driven by natural breast contours and minimal donor site morbidity, has thus bolstered its popularity [32,33].

The aim of this article is to provide an overview of the first author’s experience with SCIP/SIEA and PAP flaps in reconstructing various body regions. Initially, in our experience these flaps were used sporadically, particularly as alternatives to the DIEP flap in breast reconstruction. However, with the increasing popularity of the SCIP flap and the broader application of the PAP flap to other regions of the body [34], the author conducted an in-depth review of the literature and began applying these flaps with varying thicknesses, from full thickness to skin only. After gaining positive initial results, these flaps were systematically offered to patients, starting in 2022, to further evaluate their versatility and reliability as “workhorse” flaps.

## 2. Materials and Methods

### 2.1. Retrospective Study

This retrospective study includes all soft tissue defects requiring free flap reconstruction performed by the first author at the Department of Hand and Plastic Surgery, Cantonal Hospital of Winterthur, Switzerland, from August 2022 to August 2024. Ethical approval for the study was obtained from the Swiss Association of Research Ethics Committees (approval number: 2024-02425). Written informed consent was obtained from all patients prior to their inclusion in the study.

Patients were included if they underwent free flap reconstruction with complete follow-up data and documented postoperative records. Those receiving osteocutaneous flaps (e.g., the fibula or iliac crest) were excluded to ensure a focus on soft tissue reconstructions. From August 2022, SCIP/SIEA and PAP flaps were routinely offered for defects across all body regions, in addition to commonly used options such as RFF, mSAP, ALT, DIEP, and LD. The selection of donor sites was based primarily on patient preference. In cases where patients did not specify preferences or when specific approaches were required (e.g., two-team setups or a specific surgical positioning), the donor site was selected by the primary surgeon.

Postoperative outcomes and complications were classified by type (e.g., vascular anastomosis issues, flap loss, seroma) and severity using the Clavien–Dindo classification system. Major complications (Grades IIIa and above) included events such as flap loss requiring surgical reintervention or severe infections necessitating intravenous antibiotics. Minor complications (Grades I–II) included issues manageable through conservative measures, such as superficial infections or small areas of necrosis treated with local dressings. For patients undergoing multiple flaps, a dual analysis approach was adopted. Each patient was counted once to calculate complication rates on a per-patient basis, minimizing the overrepresentation of patients with multiple procedures. Additionally, each flap was treated as an independent data point to assess flap-specific complication rates.

### 2.2. Statistical Study

A statistical analysis was conducted to evaluate patient demographics, flap areas, and the occurrence of postoperative outcomes. Data were collected on demographic and clinical characteristics, including age, gender, and comorbidities, for all patients in each reconstruction group (SCIP/SIEA, PAP, and others). Descriptive statistics were calculated for each group to summarize patient profiles. Flap areas were analyzed descriptively to evaluate the dimensions of the SCIP/SIEA, PAP, and other flap types. Breast reconstruction cases were excluded due to missing area measurements or inconsistencies from bilateral flaps in some PAP cases. A one-way ANOVA was performed to test differences in mean flap areas between groups, with Tukey’s HSD test applied for post hoc pairwise comparisons where significant differences were identified. To compare demographic and clinical characteristics across groups, an ANOVA test was used to analyze mean ages, while Chi-square tests were applied for gender and comorbidity distributions.

All statistical analyses were conducted using Python (Python Software Foundation, Beaverton, OR, USA) version 3.10, along with the SciPy and Pandas libraries. Statistical significance was set at *p* < 0.05.

## 3. Results

### Retrospective Study Results

**Figure 3:**
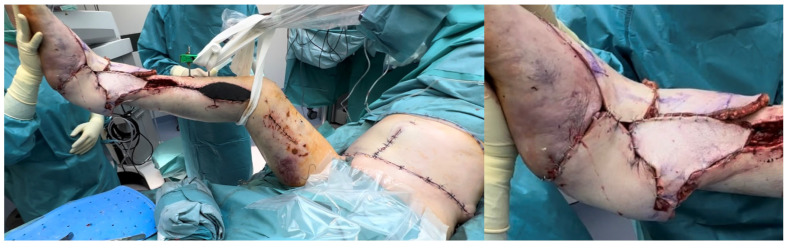
Right leg and ankle Gustilo IIIb open fracture (patient no. 11, Table S1 and no. 9, Table S3). Due to the patient’s abundant abdominal skin apron, the circumferential soft tissue defect was covered using a combination of DIEP and SCIP/SIEA flaps taken bilaterally (SCIP/SIEA from the right abdomen, DIEP from the left). Unfortunately, the operation had to be aborted due to severe cardiovascular instability, and the limb was subsequently amputated in a follow-up procedure. In such cases with wide circumferential defects, we believe a chimeric latissimus dorsi with serratus anterior would be a better option, as it provides much more efficient coverage.

A total of 79 patients with soft tissue defects underwent reconstruction with 86 free flaps. Among them, five patients received bilateral flaps (two SCIP/SIEA and three PAP), one patient received two flaps on the same side (SIEA and DIEP) (Figure 3), and one patient required a second free flap due to the failure of the initial SCIP/SIEA flap.

**Figure 4:**
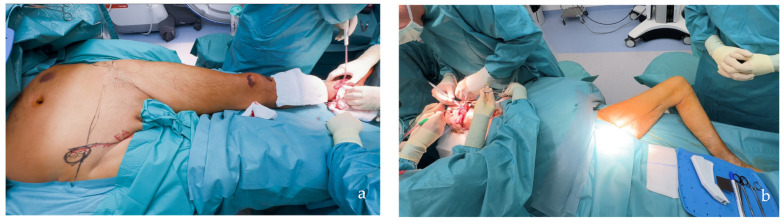
Patient positioning related to the two-teams approach. (**a**) The SCIP/SIEA flap from the lower abdomen provides an ideal harvesting position in orthoplastic cases, allowing the orthopedic surgeons to work simultaneously without interference from the other team (the same applies to head and neck cases). (**b**) The PAP flap harvested from the thigh facilitates the two-team approach, especially in head and neck cases, but is less comfortable in lower limb reconstructions where both teams are working on the lower part of the body.

**Figure 5:**
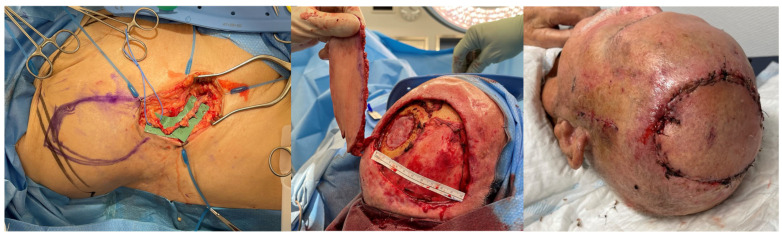
Example of scalp reconstruction. A wide SCIP/SIEA flap was transferred from the right abdomen to cover an 11 × 12 cm defect with exposed dura mater, calvarial bone, and periosteum.

**Figure 6:**
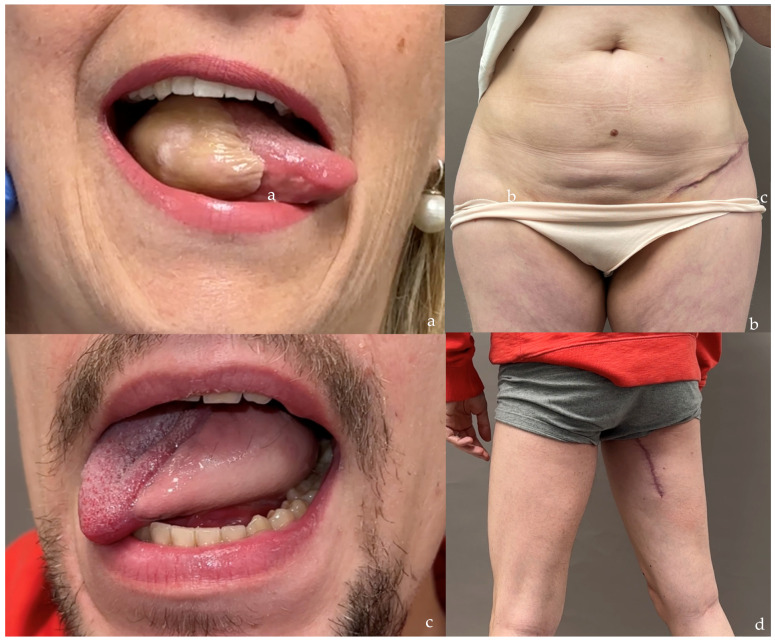
Examples of tongue reconstruction in young patients using SCIP/SIEA flaps (**a**,**b**) and PAP flaps (**c**,**d**). Both techniques result in well-concealed donor site scars. Differences in color and texture between the flaps and the native mucosa are apparent, which is typical of all skin-based free flaps.

**Figure 7:**
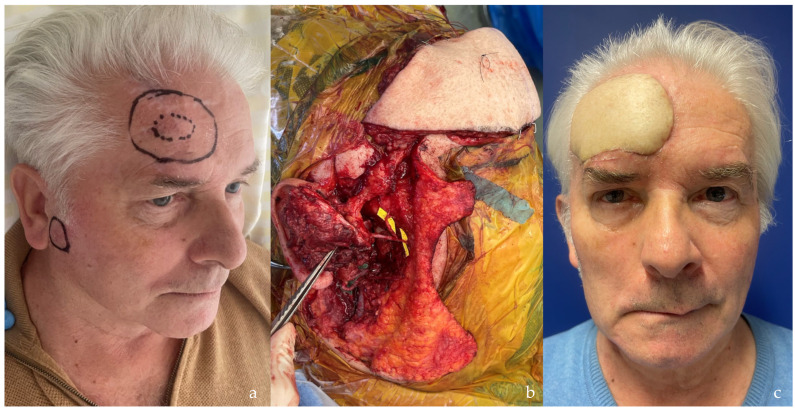
(**a**) Patient with a wide squamous cell carcinoma recurrence of the forehead following multiple previous operations. (**b**) Due to metastatic lymph nodes, a partial parotidectomy was performed, sacrificing the temporal and zygomatic branches of the facial nerve. (**c**) A chimeric musculo-cutaneous ALT flap was used to reconstruct the facial nerve gap, utilizing the motor nerve accompanying the descending branch of the lateral circumflex femoral artery (LCFA-db). A piece of vastus lateralis was used to fill the dead space after the parotidectomy. A gold weight was placed in the upper right eyelid, and a lateral canthopexy was performed to address the lagophthalmos.

**Figure 8:**
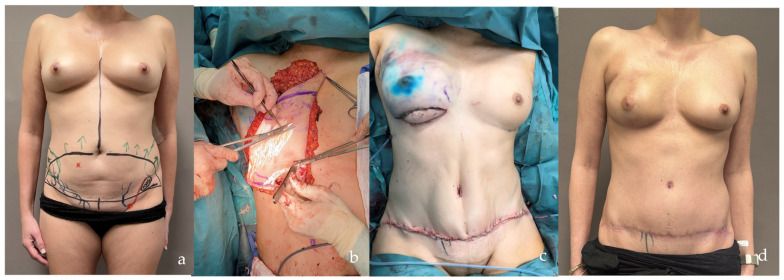
Patient with right breast cancer undergoing nipple-sparing mastectomy and SCIP/SIEA flap reconstruction. In panel (**a**), the artery is marked in red, and the SIEV in blue, on the skin of the lower left side of the flap design. The red cross marks a DIEA perforator retained as a salvage option in case of SCIP/SIEA inadequacy. In (**b**), the flap is 80% deepithelialized. Panel (**c**) shows the flap folded to provide volume and projection, with a skin island preserved for flap monitoring. Finally, (**d**) shows the result after the removal of the skin island, with a low donor site scar.

**Figure 9:**
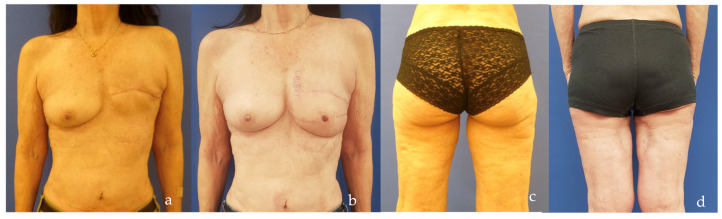
Breast reconstruction with double PAP flaps. The left breast is absent due to a previous simple mastectomy (**a**). The patient had limited abdominal tissue and consented to microsurgical reconstruction using two PAP flaps (**b**) taken from the thighs (**c**,**d**). Two smaller flaps were chosen instead of one larger flap to avoid thigh deformity and asymmetry between the thighs.

**Figure 10:**
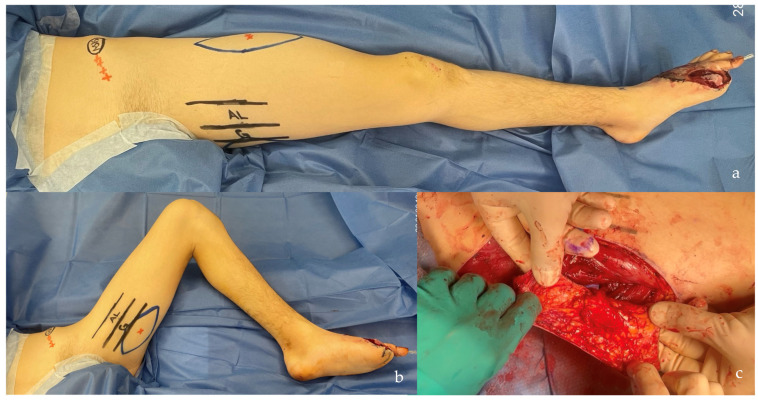
This patient suffered a traumatic amputation of the left hallux, and a free flap was planned to cover the exposed metatarsals. Considering the patient’s positioning on the operating table, three options were presented, and relative preoperative markings were performed: ALT (**a**), SCIP/SIEA (**a**), or PAP (**b**) flap. The patient chose the PAP flap. The flap was harvested as “skin only”, leaving a small hub of fat around the terminal perforator’s branching to the skin (**c**). The postoperative results are shown in Figure 11.

**Figure 11:**
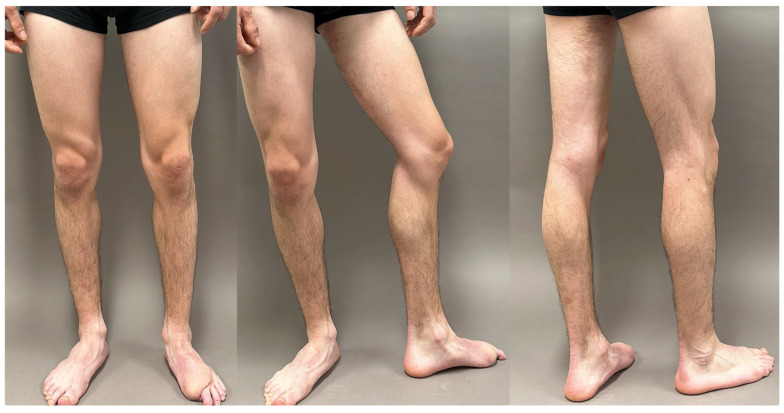
A 1.5 years postoperative follow-up of the reconstructed left foot and left PAP donor site scar of the patient shown in Figure 10. Note the good contour of the foot and the concealed donor site scar, with no need for correction.

**Figure 12:**
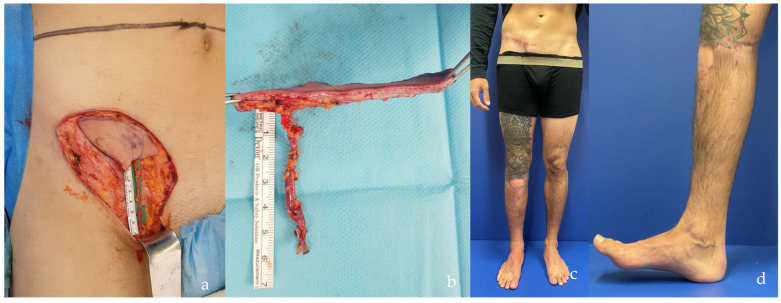
(**a**) In this patient, a SCIP/SIEA flap from the right groin region was transferred to the proximal anterior leg to cover a 7 × 5 cm defect. (**b**) The flap was harvested as thinly as possible (skin only) to provide the best contour. (**c**) The donor site is completely concealed under the panty line. (**d**) The reconstructed part shows a good contour.

**Figure 13:**
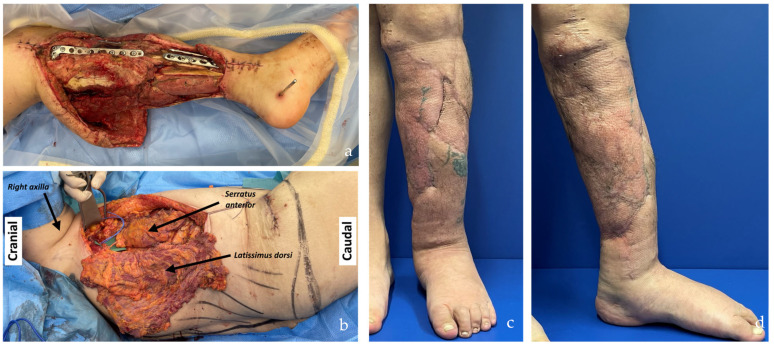
(**a**) Gustilo 3b open fracture of the left leg with a circumferential soft tissue defect. (**b**) To treat this case, a latissimus dorsi and serratus anterior free flap was transferred and skin grafted. The latissimus dorsi, alone or combined with the serratus anterior, remains the preferred (and likely the only effective) free flap for such extensive injuries. (**c**,**d**) One-year postoperative follow-up showing good contour restoration of the leg.

**Figure 14:**
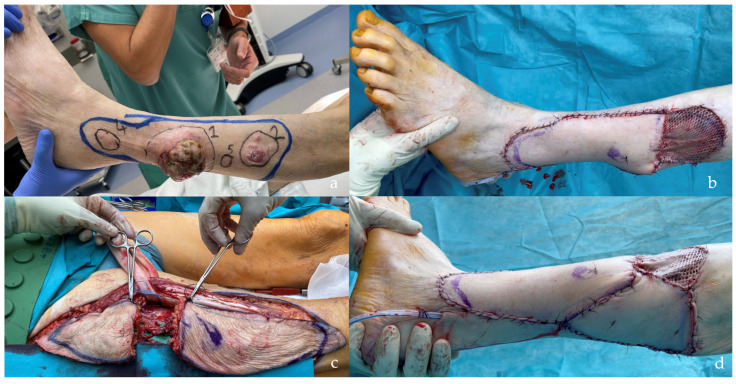
(**a**–**d**) Sarcomatoid carcinoma with multiple skin metastases involving the anterior, lateral, and posterior aspects of the left leg. Reconstruction with a chimeric two-skin island ALT flap was chosen due to the ease of harvesting a wide chimeric flap in a patient in very poor general condition.

**Figure 15:**
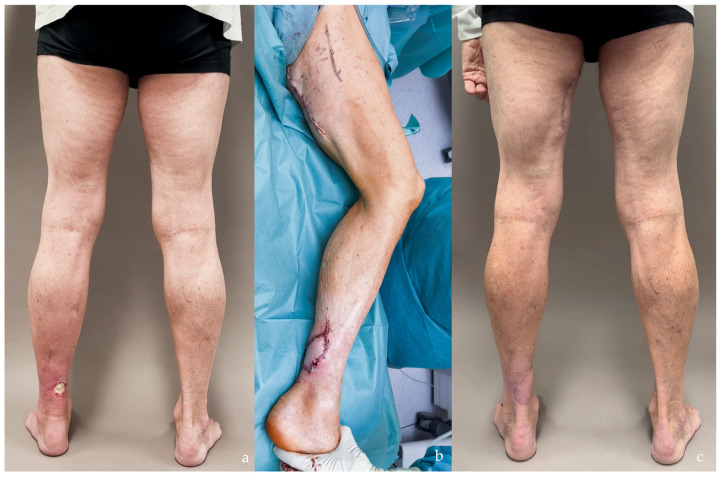
(**a**) Left Achilles tendon exposure following radiotherapy for non-melanoma skin cancer (NMSC), with local flaps deemed unreliable due to radiotherapy-induced skin changes. (**b**) A PAP flap was transferred from the ipsilateral thigh and anastomosed to the posterior tibial vessels in an end-to-side fashion. Since it was a one-team operation, the flap was harvested from the same limb. (**c**) Result after 6 months.

**Figure 16:**
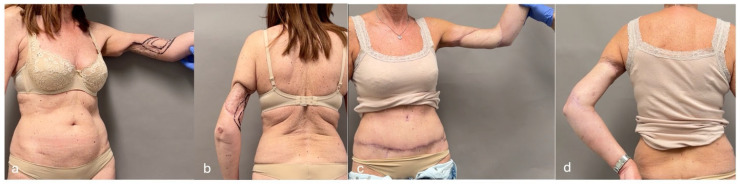
This patient suffered a sarcoma recurrence at the level of the distal third of the arm. (**a**,**b**) The elliptical resection is marked in black on the skin. Note the extensive deformity resulting from the previous surgery, involving approximately 70% of the arm circumference. (**c**,**d**) A 20 × 13 cm SCIP/SIEA flap was harvested from the right abdomen. The flap was used to reconstruct both the new and old soft tissue defects of the arm. The abdomen was closed in an abdominoplasty fashion for optimal scar concealment.

**Figure 17:**
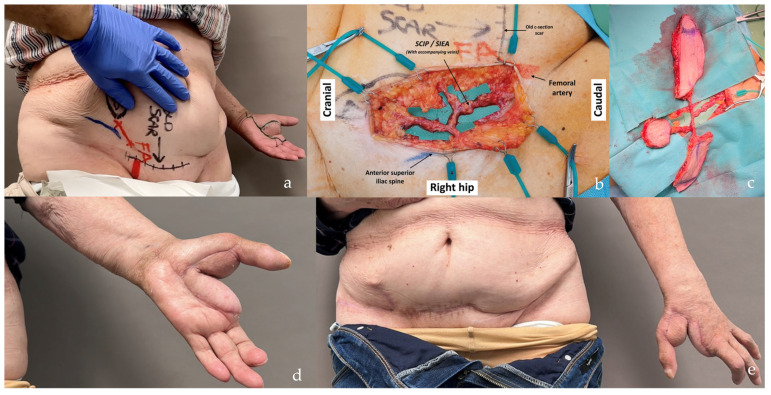
This patient suffered from a pleomorphic sarcoma on the left hand. (**a**) A wide tumor excision with the sacrifice of the second ray was planned (elliptical drawing), along with a SCIP/SIEA flap from the right abdomen. The scar from the previous C-section was marked on the skin, along with the femoral artery (FA). (**b**) The SCIP/SIEA artery (and accompanying veins) showed many branches. (**c**) Three skin islands were harvested from the available branches. The skin islands can be moved with great flexibility for a tailored reconstruction (only two of the three skin islands were used; the third was unnecessary). (**d**,**e**) Postoperative follow-up at 6 months.

**Figure 18:**
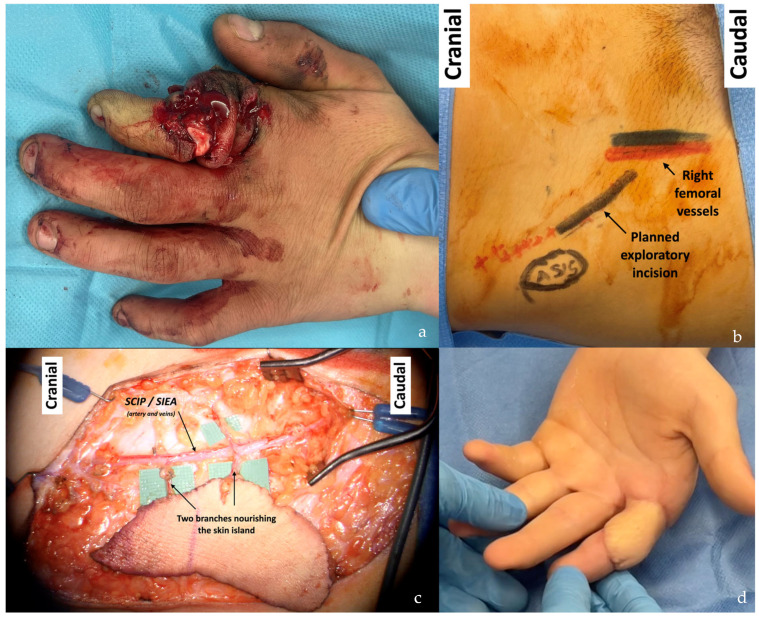
(**a**) Subtotal amputation of the second finger reconstructed with a “flow-through” SCIP/SIEA flap from the right groin. (**b**) Flap incision planning, (**c**) intraoperative flap dissection, and (**d**) 6-month postoperative follow-up. The pedicle was used to reconstruct the vascular gap of the digital vessels, and the skin island provided coverage.

The distribution of defects by anatomical region was as follows: head and neck (38.0%, n = 30) (Figure 4b, Figure 5, Figure 6 and Figure 7), breast (29.1%, n = 23) (Figure 8 and Figure 9), lower extremity (26.6%, n = 21) (Figure 3, Figure 4a, Figure 10, Figure 11, Figure 12, Figure 13, Figure 14 and Figure 15), upper extremity (5.1%, n = 4) (Figure 16, Figure 17 and Figure 18), and trunk (1.3%, n = 1). The SCIP/SIEA flap (Figure 1) was the most frequently used flap (68.4%, n = 54), predominantly for head and neck reconstruction (35.2%) (Figure 6a,b), breast reconstruction (33.3%)(Figure 8), and lower extremity defects (24.1%) (Figure 4a and Figure 12) (Table S1). The PAP flap (Figure 2) was utilized in 22.8% of patients (n = 18), mainly for head and neck (50.0%) (Figure 4b and Figure 6c,d) and breast reconstruction (27.8%) (Figure 9) (Table S2). Other flaps (e.g., ALT, LD, RFF) accounted for 8.9% (n = 7) (Figure 7, Figure 13 and Figure 14) and were primarily employed for extremity and trunk defects (Table S3). Of the 79 patients, 27 (34.2%) could choose their donor sites, with 29.6% opting for the abdomen or inner thigh. The remaining 70.4% left the decision to the surgeon.

Major complications occurred in 12 patients (15.2%), requiring surgical reintervention or intravenous antibiotics. These included flap loss, vascular compromise, dehiscence, or severe infections. Minor complications, managed conservatively, were observed in 18 patients (22.8%) and included small areas of necrosis, minor infections, and seromas. A total of four flaps (4.7% of all flaps) were lost, all of which were SCIP/SIEA flaps (7.1%). Vascular complications occurred in three flaps (3.5%), with two SCIP/SIEA flaps experiencing venous insufficiency and one PAP flap experiencing arterial occlusion, all salvaged through leech therapy or anastomosis revision. Donor site dehiscence was the most common minor complication (8.9%, n = 7), while seromas requiring aspiration were observed in six patients (7.6%). Partial flap necrosis was seen in three SCIP/SIEA cases (3.8%), managed with VAC therapy or minor surgical revision.

## 4. Statistical Study Results

### 4.1. Demographics and Clinical Characteristics

The mean age of patients in the SCIP/SIEA group was 39.4 years (SD = 20.0), compared to 48.8 years (SD = 25.1) in the PAP group and 70.0 years (SD = 17.5) in the alternative flaps group. Although age varied across the groups, the differences were not statistically significant (F = 2.75, *p* = 0.104). The gender distribution was also similar among the groups (χ^2^ = 0.60, *p* = 0.741). The prevalence of comorbidities varied but did not show a significant association with group membership (χ^2^ = 17.0, *p* = 0.523). Common comorbidities included diabetes mellitus, obesity, and smoking history in the SCIP/SIEA group, while the PAP and alternative flap groups showed higher rates of hypertension and coronary artery disease, respectively.

### 4.2. Flap Areas

The mean area for SCIP/SIEA flaps was 74.44 cm^2^ (SD = 66.09), with a range of 20–280 cm^2^. PAP flaps had a mean area of 59.77 cm^2^ (SD = 29.31), ranging from 16 to 105 cm^2^. Alternative flaps exhibited the largest mean area of 256.83 cm^2^ (SD = 189.30), ranging from 35 to 450 cm^2^. The ANOVA revealed significant differences in mean flap areas between the groups (F = 14.24, *p* = 0.000012). A post hoc analysis using Tukey’s HSD test identified significant differences between SCIP/SIEA and alternative flaps (*p* = 0.0002) and between PAP and alternative flaps (*p* = 0.0001). No significant difference was observed between SCIP/SIEA and PAP flaps (*p* = 0.75).

### 4.3. Complication Rates

Due to group differences in patient characteristics, no statistical comparisons of complication rates were conducted. However, major complications were more frequently associated with SCIP/SIEA flaps (7.1%) compared to PAP flaps (4.8%) and alternative flaps (0%). Minor complications, such as donor site seromas and dehiscence, occurred predominantly in the SCIP/SIEA group.

## 5. Discussion

Reconstructive microsurgery increasingly focuses on combining aesthetic and functional results. Recently, the number of publications on SCIP and PAP flaps has increased significantly. This surge in interest is largely due to the advantages these flaps offer in terms of scar concealment, providing more discreet scarring compared to traditionally favored workhorse flaps [16,17,18,19,20,35].

The postero-medial thigh and lower abdomen are indeed advantageous donor sites, offering both concealment and sufficient adipocutaneous tissue, which allows for direct closure even with large flaps. However, both sites also pose some challenges, particularly when harvesting thin flaps for superficial defects, as the subcutaneous layers are often thick. Despite this, the PAP flap, like the SCIP and SIEA flaps, can be harvested with varying thicknesses, making it adaptable to different reconstructive needs [36,37,38]. This versatility makes them valuable for complex cases requiring a tailored tissue thickness (Figure 10, Figure 11 and Figure 12). A summary of their advantages and disadvantages appears in Table 1.

These donor sites have also captured our interest. As early as 2018–2019, the first author began to increasingly apply SCIP/SIEA and PAP flaps in various body regions. With this initial experience and familiarity established, we sought to assess the reliability and versatility of these flaps by systematically offering them as part of our reconstructive options. Hence, they were introduced alongside the traditionally recommended flaps for soft tissue reconstruction: the radial forearm flap (RFF) and medial sural artery perforator (mSAP) flap for relatively smaller, thinner defects; the anterolateral thigh (ALT) flap for larger defects; the deep inferior epigastric artery perforator (DIEP) and transverse muscle gracilis (TMG) flaps for breast reconstructions; and the latissimus dorsi (LD) flap, either alone or combined with the serratus anterior (SA), for more extensive defects [11]. While the mSAP flap remains less commonly used globally than the radial forearm and ALT flaps, we regard it as a valuable “workhorse” flap in our practice [39].

Although the number of cases in this study is relatively limited, several valuable insights regarding the flaps used have emerged, which we believe may benefit the microsurgical community.

To accommodate patient preferences for more concealed donor sites, 34.2% of patients were offered multiple donor site options and shown visual examples during preoperative consultations (see Figure 6, Figure 8, Figure 9, Figure 10, Figure 11, Figure 15 and Figure 16). Of these, 29.6% of them selected either the SCIP/SIEA flap or the PAP flap; notably, none spontaneously preferred traditional flaps such as the RFFF, mSAP, or ALT. Among the remaining 70.4% of patients who deferred the choice to the surgeon, SCIP/SIEA or PAP flaps were still selected to build familiarity with these options and to assess their reliability and aesthetic outcomes.

Our observations indicate that, when given a choice, patients tend to prefer concealed donor sites, such as the lower abdomen or inner thigh, especially younger individuals who are more concerned with scar visibility. Patients with prominent abdomens or excess abdominal skin tended to prefer the abdomen as a donor site, likely because they already viewed it as a “cosmetic flaw” and felt an additional scar would not significantly impact its appearance. They also recognized that substantial tissue removal from this site would allow for easier closure. Notably, the SCIP/SIEA flap can improve abdominal aesthetics when the donor site is managed similarly to an abdominoplasty, removing excess tissue along with the flap (Figure 8 and Figure 16). SCIP/SIEA scars can often be placed entirely below the underwear line (Figure 6b and Figure 12); however, larger flaps may result in scars that extend onto the abdomen or flank (Figure 17).

In contrast, the PAP site was preferred by younger patients or those especially concerned about visible scarring, particularly when their abdomen was flat and lacked excess tissue. Some patients, however, expressed concerns that PAP donor site scars might cause discomfort or pain when sitting, spreading their legs, or engaging in sports like cycling. Nevertheless, in our experience, no postoperative pain or functional limitations were reported following PAP flap harvesting, aligning with the literature that suggests a low incidence of such complications [40]. Meanwhile, older patients and male patients were more likely to defer to the surgeon’s judgment, taking into account factors like surgeon preference, ease of preparation, patient positioning, and the feasibility of a two-team approach.

When considering flap reliability and success rates beyond aesthetic outcomes, it is noteworthy that although the overall flap loss rate was relatively low (4 out of 86 flaps, or 4.7% of all flaps), all instances of flap loss occurred with the SCIP/SIEA flap, resulting in a loss rate of 7.1% for this type. However, when excluding external causes of flap loss, such as pedicle damage and cardiovascular instability, the loss rate for SCIP/SIEA flaps is halved to 3.6%, closely aligning with the average reported failure rate of 2.7% in the literature [16].

Additionally, minor complications were more prevalent in the SCIP/SIEA cohort, with donor site dehiscence and seroma occurring in 10.7% and 8.9% of SCIP/SIEA flaps, respectively (compared to 4.8% for PAP flaps in both cases). This relatively high incidence of complications may also be attributable to the manipulation or damage of lymph nodes or lymphatic collectors, anatomically present in every patient at the level of the femoral triangle [41,42,43].

Furthermore, partial flap necrosis was seen in 5.6% of SCIP/SIEA flaps, whereas no cases were reported in PAP flaps. This difference in outcomes may reflect the intrinsic challenges associated with SCIP/SIEA flaps, particularly related to vessel anatomy and vascular reliability. SCIP/SIEA vessels often present with small, delicate arterial structures, and their venous drainage can be highly variable, sometimes necessitating the anastomosis of multiple veins to ensure adequate outflow (see Figure 1). 

However, when comparing the frequency of reconstructions performed with each type of flap, SCIP/SIEA flaps were used to treat a significantly higher number of defects than PAP flaps. This discrepancy can be attributed to several factors. The abdominal region typically has more excess skin, enabling the harvesting of larger and more voluminous flaps, which makes it a preferred option for breast reconstruction (see Figure 1 and Figure 8). Additionally, the abdominal donor site is conveniently positioned for surgery. This facilitates an efficient collaboration with other specialties, such as head and neck, orthopedic, and breast surgery (Figure 4). For example, in Figure 10, a young patient preferred the inner thigh donor site on the same side as his injured foot. However, the external rotation of the thigh necessary for flap preparation did not allow the simultaneous debridement and preparation of the anterior tibial vessels for anastomosis. Despite these minor drawbacks, PAP flaps demonstrated a high rate of successful reconstruction, with a very low incidence of major complications. This success makes PAP flaps a reliable option for patients requiring soft tissue reconstruction, especially for head and neck and breast defects, due to their versatility, single robust vascular pedicle (see Figure 2), and manageable donor site morbidity (Figure 6, Figure 9, Figure 11 and Figure 15).

**Figure 19:**
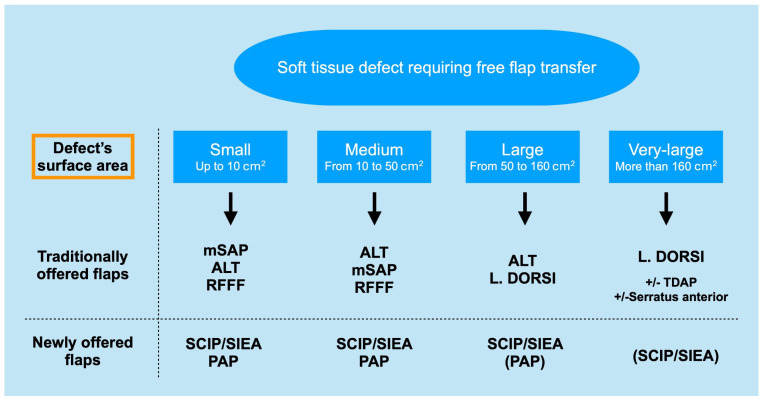
Soft tissue reconstructive options offered to patients. Based on the first author’s experience, SCIP/SIEA and PAP flaps can replace traditionally offered flaps in most cases. For small to medium-sized defects, both SCIP/SIEA and PAP flaps are suitable, and the choice of donor site can be left to the patient. As the defect size increases (large and extra-large), the abdomen typically allows for harvesting larger flaps compared to the thigh. For very large and complex defects, muscle flaps remain, in our opinion, the most efficient reconstructive option. Only in a few instances is the SCIP/SIEA flap suitable for very large defects. Breast reconstruction is not considered in this diagram. Parentheses indicate that the flap is less frequently suitable.

Additional insights into flap choice can be drawn from the analysis of flap areas, which reveals significant differences in mean sizes between SCIP/SIEA, PAP, and alternative flap types, highlighting their diversity in reconstructive suitability. SCIP/SIEA and PAP flaps exhibited similar mean areas, indicating their applicability for comparable standard reconstructions; however, SCIP/SIEA flaps showed a broader range, suggesting they can accommodate larger reconstructive needs when required (see Figure 19 detailing current reconstructive options offered to patients with soft tissue defects). For complex cases, flaps other than SCIP/SIEA and PAP exhibited notably larger and more variable areas, pointing to their suitability in extensive reconstructions where a specialized tissue volume is also required. For instance, ALT flaps were utilized to facilitate motor nerve grafts and fascia lata strips. Moreover, LD and combined LD + SA flaps provided extensive tissue requirements in large circumferential leg and foot defects (Gustilo IIIb), as well as for areas with complex surface contours (Figure 13). Chimeric flaps, which are vascularized by the subscapular artery, provide reliable vascularization and extensive coverage due to their large-caliber vessels [44]. Additionally, muscle flaps tend to adapt better to underlying bony structures as they undergo atrophy (Figure 13c,d). In contrast, harvesting skin flaps of a similar size to the latissimus dorsi and serratus anterior is technically difficult and often does not yield satisfactory results (Figure 3) [11].

We acknowledge that the retrospective design of this study inherently introduces selection bias, which may affect the generalizability of our findings. Furthermore, the absence of a control group limits the ability to draw strong comparative conclusions. We also recognize the lack of standardized or objective tools for assessing aesthetic outcomes, which would provide greater reliability and reproducibility to our results. Future studies incorporating prospective designs, control groups, and validated aesthetic assessment tools are essential to strengthen the evidence base for the use of SCIP/SIEA and PAP flaps in clinical practice.

## 6. Conclusions

In our clinical practice, the SCIP/SIEA and PAP flaps have replaced the traditionally used free flaps for small to large defects. The SCIP/SIEA flap offers the advantage over the PAP of allowing for larger reconstructions but at the cost of greater technical difficulty, primarily due to vascular variability. For very large defects, particularly in the limbs, the latissimus dorsi flap remains the most efficient and reliable solution.

## Figures and Tables

**Table 1:** Advantages and disadvantages of the SCIP/SIEA and PAP flaps compared to other flaps.

Flap Type	Advantages	Disadvantages
SCIP/SIEA Flap	- Donor Site Scar: Mostly hidden by underwear/pants. If large flaps are harvested, the donor site can be closed in an abdominoplasty fashion to enhance aesthetics (Figure 1, Figure 8 and Figure 16). - Two-Team Approach: Ideal for limb cases, head and neck (H&N) cases, and suitable for breast cases. - Dissection Type: No intramuscular dissection or muscle sacrifice needed, and no muscle relaxation required. - Flap Dimensions and Shape Versatility: Ranges from small to very large with multiple pedicle branches for chimeric, multi-lobule flaps (Figure 17). Can be used in flow-through finger replantations (Figure 18). - Pedicle Length: Long pedicle possible with centrifugal pedicle dissection (Figure 1, Figure 5 and Figure 17). - Bone Chimerism: Can include the ASIS as a vascularized bone if using the deep branch of the SCIA.	- Vessel Variability: High anatomical variability in vessel size and branching. Small, delicate arteries pose higher complication risks (Figure 12 and Figure 17). Requires more than one vein anastomosis for adequate flap perfusion with larger flaps (Figure 1). - Donor Site Morbidity: Risk of lymphatic damage with poor dissection in the femoral triangle. Potential for sensory loss in the proximal thigh if the lateral femoral cutaneous or genitofemoral nerves are transected. - Skin Color and Texture: Mismatch with surrounding areas when used for head, neck, or limb reconstruction (Figure 6, Figure 12, Figure 16, Figure 17 and Figure 18). - Flap Thickness: Harvesting thin flaps can be challenging, especially in high-BMI patients.
PAP Flap	- Donor Site Scar: Positioned in the postero-medial thigh, hidden from patient view. - Pedicle: Reliable perforator, with a long pedicle and good vessel caliber. - Two-Team Approach: Optimal harvest position for two-team cases in H&N and breast procedures. - Muscle Chimerism: Muscular branches of the pedicle can include muscle as needed.	- Flap Thickness: Technically demanding to harvest thin flaps in high-BMI patients. - Surgeon Positioning: Harvesting requires a frog-leg position, which can be uncomfortable (Figure 4). - Dissection Type: Requires intramuscular dissection and some muscle sacrifice, and intraoperative muscle relaxation may not be possible if simultaneous facial nerve monitoring is necessary (Figure 2). - Pedicle Characteristics: Usually has only one perforator reaching the skin, making multi-lobule skin flaps difficult (see comparison with ALT flap, Figure 14). - Bone Chimerism: Not possible with this flap. - Nerve Characteristics: No accompanying motor nerve, and few reports exist on flap reinnervation with sensitive nerve inclusion.

Acronyms: SIEA—superficial inferior epigastric artery, SCIA-SB—superficial circumflex iliac artery–superficial branch, H&N—head and neck, ASIS—anterior superior iliac spine, PAP—profunda artery perforator, BMI—body mass index.

## Data Availability

Dataset available on request from the authors.

## Supplementary Materials

The following supporting information can be downloaded at: https://www.mdpi.com/article/10.3390/jcm14030921/s1, Table S1: Patients reconstructed with SCIP/SIEA flap; Table S2: Patients reconstructed with PAP flap; Table S3: Patients reconstructed with soft tissue flaps other than SCIP/SIEA and PAP flaps.
