# Supplementary material for: SCIP/SIEA and PAP: The New Workhorse Flaps in Soft Tissue Reconstruction for All Body Regions
Source: J Clin Med. 2025 Jan 30;14(3):921. doi: 10.3390/jcm14030921 (PMC11818072; doi:10.3390/jcm14030921)
Supplement: Supplementary file 1 [file jcm-14-00921-s001.zip › jcm-3356711-supplementary.pdf]

Supplementary Table S1. Patients reconstructed with SCIP/SIEA flap.

Patient No.	Age	Gender	Comorbidities	Site and Cause of Defect	Flap Size (cm) and Site	Complications and Outcome
1	57	F	DM, Obesity Class 1	Right thigh; retracting scar with contour, post-traffic accident	9x7, right	None
2 *	48	F	None	Right tongue; excision of SCC	6x4, left	Numbness and paresthesia distal to the donor area (proximal thigh) with spontaneous progressive improvement (Fig. 6 c-d)
3	14	M	None	Subtotal amputation of second finger, left hand	5x4, right	Venous insufficiency in replanted finger; leeches applied; finger salvaged (Fig. 18)
4 *	22	M	None	Right heel; defect post-traffic accident	7x5, right	Venous insufficiency; flap lost, replaced with reverse sural flap
5 *	56	M	Smoker	Intraoral scarring, osteoradionecrosis after SCC treatment	6x5, left	None
6 *	71	F	Smoker	Right side of tongue; SCC	6x4	None
7	57	F	Multiple sclerosis	Distal third of right leg; soft tissue defect post-fall	7x6	Venous congestion treated with leeches; complete flap survival
8	80	M	PVD, DM, HTN, HF, Obesity Class 1	Scalp and calvarial bone defect post-SCC excision	11x12	None (Fig. 5)
9 *	75	M	DM, Obesity Class 1	Buccal mucosa; scar retraction post-cancer treatment	7x5	None
10	92	F	PVD, HTN	Left forearm; soft tissue sarcoma	17x10	None
11 ^	64	M	PVD, DM, Smoker, Obesity Class 1	Right leg and ankle; Gustilo IIIb open fracture with circumferential soft tissue defect; post-traffic accident	20x14 (SIEA + DIEP flap, see patient n.9 Table 3)	Intraoperative cardiovascular instability; procedure interrupted; limb amputation; donor site dehiscence; conservative (Fig. 3)
12 *	37	M	None	Left medial leg; Gustilo IIIb open fracture	17x7	None
13	72	F	Previous smoker	Oromandibular SCC	14x7	None
14 *	56	M	Previous smoker	Left side of tongue; SCC	7x4	None
15	43	M	Obesity Class 1	Right leg; Gustilo IIIb open fracture	10x6	None
16 * ^^	68	F	Smoker, DM, Obesity Class 2	Palate; SCC	7x4	Pedicle injury intraoperatively; new flap performed as bailout (RFFF)
17 *	65	M	PVD, Obesity Class 1	Scalp with exposed PMMA cranioplasty	9x6	Donor site dehiscence; treated conservatively
18 *	60	F	Obesity Class 2	Dorsal left foot defect	9x7	Partial flap necrosis due to venous congestion; VAC therapy and skin graft; mild sensory loss of proximal thigh; spontaneous recovery
19 *	79	M	Obesity Class 1, PVD, HTN	Osteocutaneous fistula, osteoradionecrosis	Chimeric flap with 2 skin islands; 6x4 and 7x5	None
20 *	16	M	None	Distal third of left leg; retracting scar, painful neuroma after trauma	9x5	None

21 *	84	F	PVD, HTN	Frozen neck and osteocutaneous fistula	18x7	None
22	80	F	Alcohol abuse, previous smoker, HTN	Nose; recurrent basal cell carcinoma	7x6	None
23 *	52	M	Previous smoker	Left tongue; SCC	6x5	None
24	60	F	None	Right arm; recurrent soft tissue sarcoma	20x13	None (Fig. 16)
25 *	74	F	None	Right mandible; SCC	8x4	None
26	82	F	HTN	Left hand; soft tissue sarcoma	Chimeric flap with 3 skin islands: 9x5, 7x5, 4x4	None (Fig. 17)
27	76	M	HTN, PVD	Right leg; soft tissue defect after compartment syndrome	17x6	None
28	57	M	Obesity Class 1	Right knee; Gustilo IIIa fracture	10x7	None
29	62	M	PVD	Left tibia; osteomyelitis	12x6	None
30	58	M	Smoker	Right tibia; pseudoarthrosis and osteomyelitis	13x6	Partial flap necrosis; managed conservatively with wound dressing changes
31 *	79	F	HTN, HF	Left tongue; SCC	7x4	None
32	71	F	None	Fronto-parietal; PMMA cranioplasty exposure	22x10	Donor site dehiscence; VAC therapy and skin graft; mild sensory loss in proximal thigh
33 *	46	M	None	Right maxilla; SCC	5x5	None
34 *	78	F	Previous smoker, alcohol abuse	Left mandible; osteomyelitis and osteocutaneous fistula	4x7	Infected seroma at donor site; incision and drainage performed
35	68	F	Previous smoker, PVD	Mandibular SCC recurrence	7x6	None
36	62	M	Smoker, PVD, DM	Left lateral malleolus; Gustilo II open fracture	5x4	Donor site dehiscence; VAC and secondary healing
37	54	F	Previous smoker	Left breast; primary reconstruction after NSM	Right hemi-abdominal flap	None
38	65	F	Smoker	Primary reconstruction after SSM	Left hemi-abdominal flap	Mastectomy flap necrosis; managed with debridement, VAC, and skin graft; donor site infection
39	53	F	None	Bilateral breasts; secondary reconstruction after implant failure	Left and right hemi-abdominal flaps	Seroma at abdominal donor site; outpatient aspiration required
40	57	F	Obesity Class 2, DM	Secondary reconstruction after simple mastectomy	Hemi-abdominal flap	Partial flap necrosis (10%); outpatient revision required
41	39	F	None	Right breast; primary reconstruction after NSM	Hemi-abdominal flap	Donor site dehiscence; treated conservatively (Fig. 8)
42	52	F	None	Secondary reconstruction after simple mastectomy	Hemi-abdominal flap	None
43	43	F	Obesity Class 2	Secondary reconstruction after implant failure	Left Hemi-abdominal flap	Seroma at donor site; outpatient aspiration required
44	38	F	None	Left breast; secondary reconstruction after implant failure	Left hemi-abdominal flap	Donor site wound dehiscence; outpatient revision

45	41	F	Smoker	Left breast; secondary reconstruction after implant failure	Right hemi-abdominal flap	Arterial insufficiency; flap lost; flap removal and closure with skin advancement
46	60	F	None	Right breast; primary reconstruction after SSM	Left hemi-abdominal flap	None
47	45	F	Obesity Class 1	Left breast; primary reconstruction	Right hemi-abdominal flap	None
48	41	F	Previous smoker	Secondary reconstruction after implant failure	Left hemi-abdominal flap	None
49	43	F	Active smoker	Right breast; secondary reconstruction after implant failure	Right hemi-abdominal flap	None
50	53	F	None	Right breast; primary reconstruction after SSM	Right hemi-abdominal flap	None
51	55	F	None	Right breast; primary reconstruction after SSM	Right hemi-abdominal flap	None
52	63	F	Obesity Class 2, DM, HTN	Bilateral breasts; secondary reconstruction after implant failure	Left and right hemi-abdominal flaps	Left breast wound dehiscence; treated with VAC and secondary healing; seroma treated with outpatient aspiration
53	72	F	None	Left breast; primary reconstruction after SSM	Right hemi-abdominal flap	Seroma at donor site; outpatient aspiration required
54	54	F	None	Right breast; primary reconstruction after NSM	Left hemi-abdominal flap	None

* this patient was given the possibility to chose the flap between 2 or more options.

^ same patient as Patient 9 in Table S3

^^ same patient as Patient 8 in Table S3

Acronyms: DM – Diabetes Mellitus; HTN – Hypertension; HF – Heart Failure; NSM – Nipple-Sparing Mastectomy; SSM – Skin-Sparing Mastectomy; PVD – Peripheral Vascular Disease; SCC – Squamous Cell Carcinoma; VAC – Vacuum-Assisted Closure; PMMA – Polymethyl Methacrylate; RFFF – Radial Forearm Free Flap.

Supplementary Table S2. Patients reconstructed with PAP flap.

Patient No.	Age	Gender	Comorbidities	Site and Cause of Defect	Flap Size (cm) and Site	Complications and Outcome
1 *	19	M	None	Degloving amputation, great toe (left foot), traffic accident	14 x 6	None (Fig. 10)
2	73	F	Obesity Class I, DM, HTN	Right foot, diabetic ulcer	7 x 5	Wound dehiscence, outpatient wound revision
3	38	F	Smoker	Bilateral breast implant failure	Bilateral thigh	None
4 *	77	M	None	Left Achilles tendon exposure, post-radiotherapy	4 x 4	None (Fig. 15)
5 *	37	M	None	Scar retraction post-cancer treatment	7 x 5	None
6	73	F	Smoker, COPD, HTN	Mandibular osteoradionecrosis	12 x 7	None
7 *	36	M	None	Left tongue, SCC	7 x 5	None (Fig. 6 c-d)
8 *	81	M	HTN	Left parotid region, SCC	5 x 6	Arterial thrombosis, flap salvage
9	65	F	Smoker, Obesity Class II	Left tongue and floor of mouth, SCC	11 x 6	Intraoral abscess, donor site seroma, outpatient aspirations
10 *	84	F	Previous smoker, HTN, COPD	Left tongue, SCC	7 x 5	None
11 *	28	M	Smoker	Dorsal and medial foot, Gustilo IIIa fracture, traffic accident	11 x 8	Donor site dehiscence, conservative treatment
12	75	M	HTN, COPD, CHF	Scalp defect, SCC	12 x 7	None
13	79	M	COPD	Scalp and calvarial defect, post-sarcoma excision	10 x 8	None
14 *	65	M	Smoker	Osteocutaneous fistula, mandibular osteoradionecrosis, frozen neck	15 x 7	None
15	63	F	None	Left secondary breast reconstruction post-mastectomy	Bilateral, 11 x 6	None (Fig. 9)
16	50	F	Turner syndrome	Secondary bilateral breast reconstruction	Bilateral, 11 x 6	None
17 *	38	F	None	Left breast NSM	Left thigh, 13 x 7	None
18	52	F	None	Right breast NSM	Left thigh, 12 x 6	None

* this patient was given the possibility to chose the flap between 2 or more options.

Acronyms: DM – Diabetes Mellitus; COPD – Chronic Obstructive Pulmonary Disease; CHF – Chronic Heart Failure; NSM – Nipple-Sparing Mastectomy; SCC – Squamous Cell Carcinoma; HTN – Hypertension.

Supplementary Table S3. Patients reconstructed with soft tissue flaps other than SCIP/SIEA and PAP flaps.

Patient No.	Age	Gender	Comorbidities	Site and Cause of Defect	Size of Defect (cm)	Alternative Flap Type and Reason for Choice
1	76	M	Coronary artery disease	Radical parotidectomy	12 x 7	ALT – Need for fascia lata strip for static suspension, motor nerve graft for facial nerve reconstruction
2	78	M	None	Right partial parotidectomy	7 x 5	ALT – Need for motor nerve graft for facial nerve reconstruction (Fig. 7)
3	89	M	PVD, CHF	Left leg, sarcomatoid carcinoma with multiple regional skin metastasis	Chimeric flap with 2 skin islands	ALT – Easier and faster procedure in a debilitated patient with scant abdominal tissue; need for reliable chimerism with 2 skin islands (Fig. 14)
4	64	M	Unknown	Left ankle and foot, Gustilo IIIb with circumferential defect	22 x 18	LD – Extensive and complex defect of the leg/ankle/foot
5	43	F	Obesity I	Left leg and foot, Gustilo IIIb with circumferential defect	24 x 18	LD+SA – Extensive and complex defect of the leg/ankle/foot (Fig. 13)
6	76	M	None	Soft tissue defect of the back with cervical and thoracic spine exposure and osteosynthesis exposure	16 x 9	Musculo-cutaneous LD – Operation in prone position, extensive and deep 3D defect in a thin patient, muscle required for adequate filling
7	62	M	DM, PVD, COPD	Right foot and ankle, Gustilo IIIb with circumferential defect	18 x 25	LD+SA – Extensive and complex defect of the leg/ankle/foot
8 ^^	68	F	Smoker, DM, Obesity II	Soft palate, SCC	7 x 4	RFF – Intraoperative injury of the SIEA/SCIP flap pedicle;
9 ^	64	M	PVD, DM, Smoker, Obesity I	Right leg and ankle, Gustilo IIIb open fracture with circumferential soft tissue defect (traffic accident)	18 x 11	DIEP flap combined with SIEA flap – DIEP flap from the left abdomen used in addition to the right SIEA to harvest the entire abdominal skin (Fig. 3)

^^ same patient as Patient 16 in Table S1

^ same patient as Patient 11 in Table S1

Acronyms: DM – Diabetes Mellitus; COPD – Chronic Obstructive Pulmonary Disease; CHF – Chronic Heart Failure; SCC – Squamous Cell Carcinoma; PVD – Peripheral Vascular Disease; RFF – Radial Forearm Flap; LD – Latissimus Dorsi; SA – Serratus Anterior; ALT – Anterolateral Thigh Flap; DIEP – Deep Inferior Epigastric Perforator Flap; SCIP – Superficial Circumflex Iliac Perforator Flap; SIEA – Superficial Inferior Epigastric Artery Flap.
